# Combined impacts of oregano extract and vacuum packaging on the quality changes of frigate tuna muscles stored at 3±1°C

**DOI:** 10.14202/vetworld.2019.155-164

**Published:** 2019-01-28

**Authors:** Talal Lahreche, Yilmaz Uçar, Ali Riza Kosker, Taha-Mossadak Hamdi, Fatih Ozogul

**Affiliations:** 1 Laboratory of Food Hygiene and Quality Insurance System, High National Veterinary School, Issad Abbes Avenue, Oued Smar, Algiers, Algeria; 2 Department of Biology, Faculty of Natural Sciences, Ziane Achour University, Djelfa, Algeria; 3 Department of Seafood Processing Technology, Faculty of Fisheries, Cukurova University, Adana, Turkey

**Keywords:** dipping, fish muscles, oregano extract, quality parameters, refrigerated storage, vacuum packaging

## Abstract

**Aim:**

The combined effects of oregano extract with vacuum packing (VP) on the quality enhancement of dark and white muscles of frigate tuna (*Auxis thazard*) stored as intact fillet at refrigerated (3±1°C) conditions were studied.

**Materials and Methods:**

About 35 kg of fish were filleted without skin removal and randomly divided into two groups. One group without treatment (control) and the remaining group were dipped in a sterilized oregano extract solution for 5 min. Chemical, microbiological, sensorial, and textural analyses were carried out in each of dark and white muscles of frigate tuna fillets during storage.

**Results:**

Several quality indexes were higher in dark muscle than white muscle. The sensory assessment indicated that both muscles from control had a shelf life of 12 days. Quality parameters of both muscles had the same tendency and were significantly affected by time and also by the presence of plant extract in VP. Although VP alone was sufficient to delay lipid oxidation on fish fillets, especially on dark muscle but cannot enhance the textural deterioration in both muscles.

**Conclusion:**

Consequently, the employment of such combination had a cumulative effect on preservation, resulting in prolonging the shelf life of both frigate tuna muscles.

## Introduction

Fish have a higher nutritional value since it is an excellent source of high-quality protein, vitamins, minerals, and polyunsaturated fatty acids, particularly omega-3 fatty acids [1]. *Auxis thazard*, commonly known as frigate tuna, is a small pelagic, tuna-like species, and widely distributed in worldwide. Its consumption is recently popular in many countries since it can be used as alternative raw materials for the productions of the famous tataki, surimi, and sashimi that are consumed as raw food [2]. Similar to all seafood, frigate tuna is highly perishable due to the simultaneous action of autolytic enzymes and microbial growth [3] that will be able to cause violent postmortem autolysis, thus limiting its shelf life, especially during a high feeding period when the fish contain various bacteria and enzymes in the intestinal system. Therefore, its required to develop effective methods to avoid deterioration and maintain the quality since fish are a susceptible food product and also the shelf life of fillets is shorter than for whole fish.

The shelf life of fish can be extended noticeably by modifying the environment of the product [4]. Rapid cooling or storage in crushed ice is common methods that are traditionally used to extend the shelf life of seafood products. However, in the presence of ordinary atmosphere where the growth of aerobic bacteria is stimulated, atmospheric oxygen causes undesirable intense lipid rancidity due to the high proportion of polyunsaturated fatty acids in fish flesh [5]. For this reason, lower temperatures are combined with vacuum packaging (VP) and become an increasingly widespread preservation method involving the modification of atmosphere inside the pack [4]. VP prolongs the shelf life of fish products by removing air from a low oxygen permeability pack thereby reducing the availability of O_2_ that is essential for the growth of aerobic bacteria, preventing lipid rancidity in fillets [6], ensuring correct assembly, and protection against external aggressions such as dehydration observed during the refrigeration. However, VP conditions have no important inhibiting effect on microbial growth leading to off-odors and slimy appearance of fish. Thus, its required to combine such techniques with the application of plant extract (antimicrobial) to guarantee the safety and high quality of vacuum packed products. Dipping in a natural preservative from oregano extract with vacuum packaging might provide an effective preserving system and lead to seafood with better sensory and microbiological quality than those of common packaged seafood products.

The objectives of the current work were to establish the differences in quality parameters between dark and white muscles of the fillets and to investigate the potential combined effect of oregano extract and vacuum packaging on the shelf life of frigate tuna (*A. thazard*) muscles stored as intact fillet at refrigerated (3±1°C) conditions by evaluating chemical, microbiological, sensorial, and textural parameters.

## Materials and Methods

### Ethical approval

Permission of the Animal Ethics Committee is not required to pursue such type of study.

### Plant extracts preparation

Plant materials (Oregano) were purchased from the local market and were botanically identified. Fresh aerial parts of oregano were dried in the dark at ambient temperature (<30°C). The plant materials were individually ground into a fine powder. Extraction was done in line with the method reported by Kenar *et al*. [7] with slight modifications. Ethanol from the filtrate was completely evaporated using a rotary evaporator. The extract was weighed and treated by ultraviolet-light (30 W, 50 cm irradiation distance) for 30 min. to reduce the naturally existing microflora. Then, the extract was re-dissolved in a small volume of absolute ethanol and stored in amber flasks in the dark at −18°C till usage.

### Fish sample preparation

About 35 kg of frigate tuna (*A. thazard*) caught from the Mediterranean Sea in Turkey were purchased from the local fish market; they were delivered to the laboratory in <6 h post-capture. On arrival at the laboratory, fish were promptly gutted, decapitated, and filleted without skin removal. After that, fillets were rinsed with tap water and were randomly divided into two groups. One group kept as control, and the remaining group was dipped in a 1 L of sterile purified water consisting of 5 g of sterilized oregano extract for 5 min. The applied concentration (0.5%) of oregano extract was not perceptible by the sensory panel.

### Packaging materials

Untreated (control) and treated groups were packaged (three fillets/package) in bags of polyamide film (Polinas, Manisa, Turkey) using a Reepack RV50 vacuum packaging machine (Seriate, Via dell’Artigianato, Italy). The thickness of the bags was 90 µm while permeability of water and oxygen was 8.5 g m^−2^ 24 h and 160 cm^3^ m^−2^ 24 h, respectively.

### Storage and sampling

All fillets were kept at refrigerated (3±1°C) conditions. Three packs (nine fillets) from each treatment group were analyzed at each sampling days. Chemical, sensory, microbiological, and texture measurements were done in triplicate from separated dark and white muscles of the same fillet on days 0, 4, 8, 12, 15, and 18.

### Sensory analysis

The quality index method (QIM) was employed for frigate tuna fillets with slight modifications for sensory assessment [8]. The QIM procedure contained five quality parameters including surface and skin appearance, odor, texture and color of fillet, and plant extract odor. The scheme generally has a score system of 4 demerit points for each of the parameters. QIM assigns a score of zero for a very fresh fillet while an increasingly greater score as the fillet deteriorates. The panel, consisting of six trained evaluators, inspected fillets and recorded the appropriate demerit point for each of parameters and then the scores of all parameters are summed to give an overall demerit score, so-called quality index score. The panel was asked to indicate whether or not frigate tuna were acceptable for the assessment of the shelf-life of fish fillets.

Sensory evaluations of cooked frigate tuna fillets were assessed in line with the same method of Ariyawansa *et al*. [8] without any modification. Frigate tuna fillets were microwaved in for 3 min. (500 W) and then evaluators were asked to evaluate it. Evaluators scored for odor and flavor, using a Torry score with eight-point hedonic scale (3, poorest quality to 10, best quality).

### Chemical analysis and pH

pH values were recorded using the method proposed by Woyewoda *et al*. [9]. The total volatile basic nitrogen (TVB-N) content was analyzed in line with the method of Antonacopoulos [10]. Free fatty acid analysis (FFA) was carried out in line with the American Oil Chemists’ Society (AOCS) [11]. The thiobarbituric acid reactive substances value (TBARs) was determined in relation to the method of Tarladgis *et al*. [12] to measure the oxidation stability. Peroxide value (PV) was done by AOCS [11] method.

### Microbiological analysis

Preparation of test samples from frigate tuna muscle, initial suspension and serial decimal dilutions for microbiological analysis were prepared using sterile quarter-strength Ringer’s solution (Fluka; Sigma-Aldrich, Sweden) [13]. Aliquots of 0.1 ml from each dilution were carefully spread over the surface of the dry plate count agar (Fluka 70152; Steinheim, Switzerland) [14,15]. The enumeration of aerobic mesophilic and psychrophilic bacteria was performed after incubation for 72 h at 30°C [14] and for 10 days at 6.5°C [15], respectively. All analysis was made in triplicate and was taken from each of dark and white muscles of each of two different groups.

### Texture profile analysis (TPA)

Measurements of textural changes were carried out in line with the modified procedure of Liu *et al*. [16]. TPA was measured by a TA.XT Texture Analyzer equipped with a load cell of 50 N and the software was Texture Expert, v1.20 (Stable Micro Systems, Godalming, Surrey, U.K). Dark and white muscles were sampled from the same frigate tuna fillet with a length of 35 mm, a height of 20±2 mm, and the thickness of 10 mm. All samples were dried with filter paper after treatment. A flat-ended cylinder (P/36R) used with a diameter of 35 mm was pressed into the fillet at a constant speed of 1 mm/s until it accomplished 50% of the fillet thickness. Afterward, the force was lessened, and the sample was permitted to recover 10 s with the cylinder just contacting the sample surface. Finally, the probe was forced second on fish muscles, and the values of the hardness, chewiness, adhesiveness, resilience, and springiness were taken for the dark and white muscle.

### Statistical analysis

Average values and standard deviations were obtained from triplicate data for each of muscle type from each treatment. Data were assessed using analysis of variance. Duncan’s multiple range tests were followed to determine the significant differences at p<0.05. Statistical assessment was done by the Statistical Package for the Social Sciences (SPSS 19.0 for Windows, SPSS Inc., Chicago, IL, USA).

## Results and Discussion

### Sensory assessments

**Figure-1:**
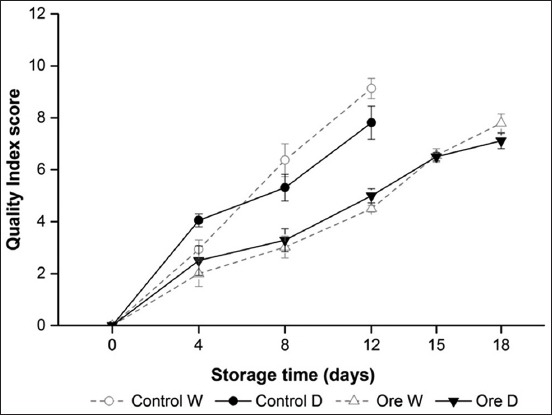
Sensory assessment of raw dark (D) and white (W) muscle from frigate tuna fillet during the refrigerated storage under vacuum pack: Untreated (Control), Oregano extract (Ore). Mean scores of sensory assessment (n=6). Standard deviations are indicated by bars.

Sensory assessment of raw and cooked dark and white muscles from frigate tuna fillets stored under VP with and without oregano extract was evaluated throughout 18 days of refrigerated (3±1°C) storage (Figure-1). No significant differences in sensory assessment (p>0.05) were observed between the two muscles type of each treatment. Quality parameters (i.e., brightness, texture, odor, and color) of both muscles in control were poorer than those of the treated group. Using a sensory score of 8 as the limit of acceptability, the shelf life was found to be no longer than 12 days for both dark and white muscles of control. The application of oregano extract extended the shelf life of white muscle by 6 additional days compared with untreated sample counterpart and maintained the acceptability of dark muscle until the end of the storage. Therefore, oregano extract extended the shelf life of both muscles of frigate tuna fillets and resulted in an improvement in sensory quality on dark muscle than on white muscle. The observed shelf life extensions of fish muscles may be resulted from the cumulative impacts of the combined treatments of filleting, VP and plant extract during the refrigerated storage.

**Figure-2:**
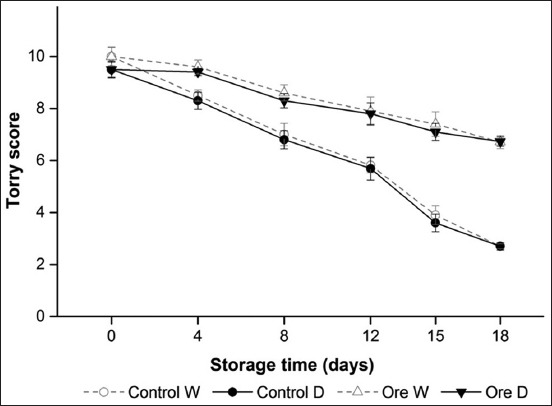
Sensory score of cooked dark (D) and white (W) muscle from frigate tuna fillet during refrigerated storage under vacuum pack: Untreated (control), oregano extract (Ore). Mean scores of sensory assessment (n=6). Standard deviations are indicated by bars. The limit of acceptability was defined by panelists as score of >6.

Sensory scores of cooked frigate tuna fillets stored under VP with and without oregano treatment dropped during the 18 days of refrigerated (3±1°C) storage (Figure-2). Throughout the experiment, no significant differences (p>0.05) were observed between both muscles for each treatment. However, significant differences (p<0.05) were observed between control and treated groups in each muscle type. Treated group was highly preferred by panelists due to their desirable flavor. The limit of acceptability was defined by panelists as a score of >6. As spoilage progressed, the sensory quality of dark and white muscle decreased until they were no longer edible at 12 days for control. However, the limit of acceptability was not reached until the 18^th^ day of the storage in both dark and white muscles for treated sample. At the rejection points, general acceptability scores of cooked frigate tuna fillets were in agreement with their raw samples corresponding of each type of muscle. As a result, it can be concluded that combined treatment could be used to extend the shelf life of frigate tuna muscles.

### pH and chemical analysis

pH values for all samples are given in Table-1. The initial pH value of dark muscle was significantly (p<0.05) higher than that of white muscle. This result was not consistent with those reported by Chaijan *et al*. [2] for the same studied species, suggesting that pH can vary not only between white and dark muscle but also in the same species, which might have been due to the variety of factors including season, harvesting procedures, biological condition, and methods of killing [17]. Throughout the storage period, pH values of dark muscle were significantly (p<0.05) higher than that of white muscle. This situation can be explained by the formation of lactic acid [18], resulting from the depletion of glycogen as a major source of energy under anaerobic conditions due to the intense activity produced in white muscle when catching [19] and leading to a decrease in the pH values of these muscles compared to the dark muscle. Regardless of the treatment, changes in pH values had the same tendency in both muscles during the storage time. However, significant (p<0.05) increases followed by a decrease in pH values were observed until days 12 and 15 for oregano and control samples, respectively. The increase in pH may be attributed to the accumulation of alkaline compounds such as ammonia and trimethylamine mainly produced by fish spoilage bacteria and/or to biogenic amines production [20]. The decrease in pH value may be caused by the anaerobic decomposition of glycogen in fish [21]. Comparison among treatments showed that fillets from control developed a higher (p<0.05) pH value than their corresponding individuals from treated samples at days 8, 15, and 18 for both muscles. No differences (p>0.05) were observed between treatment at days 4 and 12 in both muscles. At the end of the storage, pH values of control were significantly (p<0.05) lower than those of treated sample, which might have been caused by producing lactic acid from the growth of lactic acid bacteria [18]. From these results, it is difficult to conclude that the application of oregano in combination with VP can affect the pH value. Thus, pH value is not useful as a physicochemical parameter of fillet quality.

**Table-1:** Effect of oregano extract with vacuum-packaging on pH, TVB-N, and PV in white (W) and dark (D) muscles of frigate tuna fillets stored at refrigerated (3±1°C) conditions.

Storage time (days)	Parameters	pH (n=3)		TVB-N (n=3) (mg per 100 g)		PV (n=3) (meq O_2_/kg)	
			
	Samples	Control	Oregano	Control	Oregano	Control	Oregano
0	W	5.69±0.01a,Y	5.69±0.01a,Y	12.82±0.41a,Y	12.82±0.41a,Y	2.61±0.23a,X	2.61±0.23a,X
	D	5.77±0.02a,X	5.77±0.02a,X	17.21±0.84a,X	17.21±0.84a,X	1.45±0.20a,Y	1.45±0.20a,Y
4	W	5.73±0.04a,Y	5.71±0.21a,Y	18.94±0.70a,Y	18.39±2.33a,X	3.83±0.04a,X	2.60±0.73b,X
	D	5.89±0.06a,X	5.85±0.09a,X	28.29±0.88a,X	22.37±3.54b,X	1.88±0.07a,Y	1.91±0.19a,X
8	W	5.85±0.04a,X	5.77±0.06b,Y	21.41±0.73a,Y	19.84±0.35b,X	6.33±1.15a,X	2.00±0.33b,X
	D	5.89±0.03a,X	5.81±0.01b,X	22.96±0.63a,X	19.80±0.56b,X	0.99±0.02a,Y	1.03±0.00a,Y
12	W	5.87±0.02a,Y	5.86±0.07a,X	19.59±0.04a,X	15.21±0.77b,Y	3.23±0.26a,X	2.06±0.35b,X
	D	5.93±0.02a,X	5.89±0.06a,X	19.25±0.20b,Y	20.96±0.73a,X	1.62±0.01a,Y	0.94±0.02b,Y
15	W	5.88±0.02a,X	5.76±0.01b,Y	30.65±0.07a,X	25.92±0.64b,X	4.37±0.99a,X	2.57±0.08b,X
	D	5.92±0.02a,X	5.85±0.02b,X	30.11±0.28a,Y	25.88±0.69b,X	5.32±0.25a,X	2.55±0.09b,X
18	W	5.76±0.02b,Y	5.89±0.04a,X	26.80±0.71a,Y	23.39±1.23b,X	5.27±0.65a,X	2.07±0.05b,X
	D	5.82±0.02b,X	5.92±0.02a,X	29.31±0.52a,X	24.21±0.22b,X	2.16±0.04a,Y	2.03±0.16a,X

a,b=Different lowercase letters in the same row indicate significant differences (p<0.05) between samples in the same muscle during. X,Y=Different capital letters in the same column indicate the significant difference (p<0.05) between two muscle in the same sample type. TVB-N=Total volatile basic nitrogen

The variation of TVB-N for both muscles of frigate tuna fillets is shown in Table-1. TVB-N formation was reported to increase with time of storage [22]. The initial TVB-N values were significantly lower (p<0.05) in white muscle than in dark muscle. These values are compatible with those reported by Liu *et al*. [16] for skipjack tuna (*Katsuwonus pelamis*). In this study, TVB-N values showed a trend to increase with storage time in both muscles for all packed samples until 15 days, followed by a decrease which could denote the low bacterial activity at the end of storage period. The rate of increase in TVB-N in both muscles was in accordance with its rate of increase in mesophilic and psychrophilic bacteria. The statistical analysis showed significant (p<0.05) differences between dark and white muscles of fillets from control, while no significant (p>0.05) differences were observed between both muscles for oregano treated samples at each day of testing. Throughout the storage period, TVB-N values of control were significantly higher (p<0.05) in dark muscle than in white muscle, indicating that dark muscle spoiled faster than white muscle. However, in both muscles, treated samples produced significantly lower (p<0.05) TVB-N values as compared to control. The significant reduction of TVB-N values in treated samples may be attributed to the combined effect of VP with the antibacterial properties of the phenolic compounds of oregano extract such as carvacrol and thymol [23]. Liu *et al*. [16] reported that 30 mg of nitrogen per 100 g was the limit of acceptability for fresh fish fillet. Only muscles from control exceeded this limit on the 15 days of storage. However, TVB-N values of treated sample were below this limit until the end of the storage and contained lower values. Thus, TVB-N may be considered as a suitable quality index for filleted frigate tuna stored at 3±1°C under VP.

PV was used for determining the primary product of lipid oxidation [24]. Initial PV of filleted frigate tuna kept under vacuum condition at refrigerated storage was 1.45 and 2.61 meq O_2_/kg of fat for dark and white muscles, respectively. Sohn and Ohshima [25] reported that the levels of lipid peroxides of separated skipjack tuna muscles were higher in dark muscle compared to white muscle throughout the 72 h of ice storage. In addition, Dean [26] reported that dark muscle has a considerably higher capacity for fatty acid oxidation than the white muscle *in vitro* experiments. Contrary to this and during the almost entire storage period (Table-1), PV in white muscle was significantly (p<0.05) higher than in dark muscle and did not exceed the pre-established limit of 20 meq O_2_/kg of fat in all packaged samples throughout the storage period. This may be attributed to the use of intact fillets and VP because in intact fillet, skin and white muscles detain almost the entire surface that comes into contact with air and provides a protective environment for the dark muscle, which means that white muscle has more surface area that comes into contact with oxygen. Thus, white muscles consume a great deal of oxygen and thus prevent it from reaching to the dark muscle; hence, the oxygen availability on the surface area of white muscle is superior, making it much more exposed to lipid oxidation than dark muscle for fillet. This is supported by Undeland *et al*. [27] who reported that dark muscle stored within an intact herring fillet presented lower lipid oxidation when it is stored separated from skin and white muscle. In addition, VP involved complete removal of oxygen with hermetic sealing and prevented fillet from being in direct contact with O_2_ resulting in a significant reduction on lipid oxidation during the storage [28].

In this study, VP with plant extract reduced peroxide formation (p<0.05) in both muscles. The used plant extract in combination with VP is effective to delay lipid peroxidation in both muscles of frigate tuna fillets stored at refrigerated temperature. On the other hand, in the dark muscle, no difference (p>0.05) was observed until 8 days of the storage among all treatments. This stability in dark muscle may be related to the use of intact fillet. From those results, it can be concluded that the major effect on reducing PVs is attributed to the use of VP. However, the presence of oregano extract in the VP led to a further reduction of lipid peroxidation and yielded the lowest PV in both types of fish muscles.

The presence of TBARs is a second breakdown product resulting from the decomposition of lipid hydroperoxides formed during the oxidation process of polyunsaturated fatty acids [29]. Higher (p<0.05) initial TBA values were found in dark muscle than in white muscle (Table-2), which might be due to higher lipid content and unsaturated fatty acids in dark muscle. The lower initial TBA values observed in both muscles suggested that secondary lipid oxidation in our fresh fish did not occur during the postmortem handling. Subsequently, from 4 days of storage, TBA content in white muscle was significantly (p<0.05) higher than in dark muscle throughout the storage period. These changes in TBA values suggested that hydroperoxide decomposition rate was faster in white muscle than in dark muscle, which might be due to the use of intact fillet with the removal of oxygen. In the present study, TBA values in all samples showed fluctuation in both muscles; there is a trend toward an increase in TBA values up to a certain point during the storage period, followed by a decrease in these values, which is not stable for long periods of time. Practically at each testing day, and regardless of muscle type, higher TBA value (p<0.05) was observed in control compared to treated samples. Nevertheless, an inverse result was observed only in dark muscles from control and oregano samples on day 4. Various limits of acceptability were reported for this index. According to Fan *et al*. [30], TBA values of 1-2 mg 3,4-Methylenedioxyamphetamine (MDA)/kg of fish flesh are usually regarded as the limit beyond which fish normally develop an objectionable flavor. In our study, TBA values exceeded 1 mg MDA/kg in both muscles from control at 12 days of storage.

**Table-2:** Effect of oregano extract with vacuum-packaging on TBA and FFA in white (W) and dark (D) muscles of frigate tuna fillets stored at refrigerated (3±1°C) conditions.

Storage time (days)	Parameters	TBA (n=3) (mg MDA/kg)		FFA (n=3) (% oleic acid)	
		
	Samples	Control	Oregano	Control	Oregano
0	W	0.56±0.01a,Y	0.56±0.01a,Y	3.73±0.05a,Y	3.73±0.05a,Y
	D	0.64±0.01a,X	0.64±0.01a,X	11.54±0.15a,X	11.54±0.15a,X
4	W	0.84±0.04a,X	0.83±0.02a,X	6.55±0.30a,Y	5.45±0.06b,Y
	D	0.57±0.02b,Y	0.64±0.03a,Y	9.47±0.83a,X	6.96±0.20b,X
8	W	0.91±0.06a,X	0.78±0.06b,X	7.80±0.17a,Y	5.75±0.40b,Y
	D	0.66±0.01a,Y	0.65±0.01a,Y	18.83±0.90a,X	14.43±0.52b,X
12	W	1.46±0.01a,X	0.84±0.02b,X	8.28±0.24a,Y	6.61±0.19b,Y
	D	1.12±0.01a,Y	0.77±0.01b,Y	15.65±0.83a,X	12.75±0.41b,X
15	W	0.89±0.01a,X	0.58±0.01b,X	8.42±0.03a,Y	7.61±0.18b,Y
	D	0.63±0.02a,Y	0.55±0.01b,Y	18.25±0.06a,X	13.99±0.11b,X
18	W	1.28±0.02a,X	0.85±0.02b,X	10.30±0.04a,Y	7.51±0.01b,Y
	D	1.31±0.01a,X	0.72±0.12b,Y	20.53±0.37a,X	15.75±0.02b,X

a,b=Different lowercase letters in the same row indicate significant differences (p<0.05) between samples in the same muscle. X,Y=Different capital letters in the same column indicate significant difference (p<0.05) between two muscle in the same sample type. TBA=Thiobarbituric acid, MDA=3,4-Methylenedioxyamphetamine, FFA=Free fatty acid

The installation of lipid oxidation and accumulation of oxidative products can be delayed using natural extract. However, it is necessary to account on that muscle type, removal of oxygen and plant extract application along with other factors that can affect fat content in fish, will contribute to the variation of MDA content in fisheries products and must have been considered during storage. As a consequence of TBA-results, it can be concluded that the use of oregano extract in combination with VP was effective in retarding TBA formation in both muscles of frigate tuna fillets and can contribute to enhancing oxidative stability and to obtain lower TBA values throughout the storage period.

FFA, indicator of hydrolytic activity, may be accumulated during storage and accelerate quality deterioration of seafood products [31]. The release of FFA may enhance lipid oxidation and off-flavor development and indirectly cause textural proprieties by protein denaturation which is associated with a loss of freshness [32]. In our study, FFA concentration increased with storage time (Table-2) and could be attributable to the lipase and phospholipase activity [17]. Throughout the storage period, and irrespective to the use or not of plant extract, lipid hydrolysis was faster (p<0.05) in dark muscle than white muscle. This might be due to the greater lipid content and enzymatic activity presented in dark muscle than in white muscle. However, noticeable decreases followed by an increase were observed only in dark muscle at days 4 and 12, which could be related to the increased lipid oxidation, to the interaction with proteins and/or could be in junction with the growth of some microorganisms using FFA as an energy source [33]. On the other hand, at each testing days, lipid hydrolysis development, in both white and dark muscles, was significantly affected by plant extract application and was higher (p<0.05) in control than in treated samples. These results might be attributed to the effect of phenolic compounds of oregano extract on which inhibit enzymatic action liberating FFA. In addition, the use of the protective effect of oregano extract in combination with VP had a synergetic effect on retarding FFA formation of fish muscles.

### Microbiological assessments

The progress of microbial growth in frigate tuna muscles during refrigerated storage under vacuum pack for all treatments is shown in Table-3. The initial loads of mesophilic bacteria counts of vacuum-packed fillets were significantly (p<0.05) lower in white muscle than that in dark muscle. Similar results were reported by Mbarki *et al*. [34] without mentioning a specific site sampling of Atlantic bonito (*Sarda sarda*) fillet. The lower initial microbial counts obtained from this study indicated that the fish used was of good quality and might be due to the effect of washing with tap water when filleting. The microbial counts increased significantly (p<0.05) with storage time for all samples for both muscles. During the storage time, statistical analysis indicated that mesophilic bacteria were not affected (p>0.05) by the muscle type in all treatments, even in control, probably due to the exclusion of oxygen from the pack. However, mesophilic bacteria grew more quickly in control than in treated samples, indicating an antibacterial effect of the used plant extract on fish fillets.

**Table-3:** Effect of oregano extract with vacuum-packaging on the mesophilic and psychrophilic bacterial counts in white (W) and dark (D) muscles of frigate tuna fillets stored at refrigerated (3±1°C) conditions.

Storage time (days)	Samples	Mesophilic aerobic bacteria (n=3) (log CFU/g)		Psychrotropic bacteria (n=3) (log CFU/g)	
	
		Control	Oregano	Control	Oregano
0	W	2.95±0.16a,Y	2.59±0.16a,Y	2.95±0.06a,X	2.95±0.06a,X
	D	3.31±0.15a,X	3.31±0.15a,X	2.86±0.10a,X	2.86±0.10a,X
4	W	3.55±0.08a,X	2.79±0.43b,X	3.74±0.31a,X	3.19±0.15b,X
	D	3.82±0.20a,X	3.31±0.21b,X	3.56±0.32a,X	3.32±0.36a,X
8	W	3.89±0.36a,X	3.35±0.32b,Y	4.12±0.12a,X	3.32±0.28b,X
	D	4.01±0.06a,X	3.87±0.19a,X	3.89±0.16a,X	3.59±0.25a,X
12	W	5.07±0.23a,X	4.36±0.31b,X	4.85±0.42a,X	4.15±0.21b,X
	D	4.93±0.13a,X	4.36±0.33b,X	4.64±0.30a,X	4.02±0.05b,X
15	W	7.05±0.04a,X	5.57±0.10b,X	5.75±0.21a,X	5.36±0.32a,X
	D	6.74±0.38a,X	5.11±0.12b,Y	6.04±0.08a,X	5.10±0.17b,X
18	W	7.39±0.17a,X	5.43±0.24b,X	5.93±0.10a,X	5.39±0.07b,X
	D	7.62±0.14a,X	5.29±0.11b,X	6.01±0.23a,X	5.56±0.09b,X

a,b=Different lowercase letters in the same row indicate significant differences (p<0.05) between samples in the same muscle. X,Y=Different capital letters in the same column indicate the significant difference (p<0.05) between two muscle in the same sample type, CFU=Colony-forming unit

Psychrotrophic bacteria counts were higher than mesophilic bacteria counts indicating that psychrotrophic bacteria constituted the dominant bacterial flora in fish. During the storage period, no significant difference (p>0.05) was observed between psychrotrophic bacteria counts of white and dark muscles. However, psychrotrophic bacteria counts in white and dark muscle from the treated sample were lower than control through the most of storage time, indicating antibacterial effects of oregano extract on fish fillets.

Both mesophilic and psychrotrophic bacteria counts exceeded 6 log_10_ colony-forming unit/g in both muscles from only control at 15 days of the storage. These results indicated that the combination of filleting, antibacterial properties of phenolic compounds of the used plant extract and the removal of oxygen from pack had a beneficial effect on reducing the growth of mesophilic and psychrotrophic bacteria during refrigerated storage, which may be attributed to the exclusion of oxygen that inhibited the growth of many microorganisms. On the other hand, the smaller size of fillet cooled faster than the whole fish. In addition, filleting increased the surface area and contact with the cold air as well as phenolic compounds. Compared with control, regardless of the muscle type, a microbiological shelf life extension that is more than 3 days was achieved for treated samples with oregano extract, as stated previously by sensory assessments and TVB-N.

### TPA

The results of TPA of treated and untreated frigate tuna fillets are presented in Table-4 and -5. TPA is the most important attribute for assessing fish freshness that reproduces the jaw action and is influenced in the same species by several factors including size and age of fish, fat content, muscle fibre density, slaughter method, and postmortem factors such as rigor mortis, proteolysis, microbiologic, and storage conditions [35,36].

**Table-4:** Effect of oregano extract with vacuum packaging on the hardness, the springiness and the adhesiveness of white (W) and dark (D) muscles of frigate tuna fillets stored at refrigerated (3±1°C) conditions.

Storage time (days)	Parameters	Hardness (n=3) (N)		Springiness (n=3) (ratio)		Adhesiveness (n=3) (N. s)	
			
	Samples	Control	Oregano	Control	Oregano	Control	Oregano
0	W	45.17±0.05a,X	45.17±0.05a,X	1.90±0.03a,Y	1.90±0.03a,Y	0.21±0.03a,Y	0.21±0.03a,Y
	D	45.02±0.11a,X	45.02±0.11a,X	1.97±0.02a,X	1.97±0.02a,X	0.43±0.02a,X	0.43±0.02a,X
4	W	44.49±0.37b,X	45.08±0.05a,X	1.59±0.03a,Y	1.65±0.14a,X	0.12±0.03a,Y	0.16±0.02a,Y
	D	43.11±0.10b,Y	43.94±0.10a,Y	1.98±0.01a,X	1.75±0.04b,X	0.31±0.03b,X	1.29±0.06a,X
8	W	40.08±0.81b,X	42.22±0.08a,X	1.70±0.02a,X	1.72±0.04a,X	0.11±0.04b,Y	1.17±0.03a,X
	D	38.14±0.08b,Y	39.14±0.05a,Y	1.70±0.02a,X	1.71±0.03a,X	1.19±0.02a,X	1.25±0.04a,X
12	W	38.76±0.28b,X	41.06±0.05a,X	1.62±0.05b,X	1.75±0.06a,X	1.07±0.05a,X	1.09±0.02a,X
	D	37.56±0.50b,Y	39.14±0.15a,Y	1.67±0.02a,X	1.71±0.02a,X	1.13±0.04a,X	1.14±0.06a,X
15	W	35.29±0.26b,X	40.03±0.11a,X	1.53±0.03b,X	1.64±0.03a,X	1.03±0.05a,X	1.12±0.05a,X
	D	33.28±0.14b,Y	35.45±0.27a,Y	1.63±0.07a,X	1.65±0.02a,X	1.07±0.02b,X	1.17±0.02a,X
18	W	30.28±0.24b,X	35.84±0.18a,X	1.42±0.04a,Y	1.53±0.14a,X	0.90±0.10a,X	0.83±0.02a,X
	D	28.05±0.21b,Y	31.65±0.31a,Y	1.57±0.02a,X	1.48±0.02b,X	1.05±0.04a,X	0.90±0.04b,X

a,b=Different lowercase letters in the same row indicate significant differences (p<0.05) between samples in the same muscle. X,Y=Different capital letters in the same column indicate the significant difference (p<0.05) between two muscle in the same sample type. TPA=Texture profile analysis

**Table-5:** Effect of oregano extract with vacuum packaging on the resilience and the chewiness of white (W) and dark (D) muscles of frigate tuna fillets stored at refrigerated (3±1°C) conditions.

Storage time (days)	Parameters	Resilience (n=3) (ratio)		Chewiness (n=3) (N)	
		
	Samples	Control	Oregano	Control	Oregano
0	W	0.003±0.001a,X	0.003±0.001a,X	1.15±0.04a,X	1.15±0.04a,X
	D	0.001±0.000a,Y	0.001±0.000a,Y	1.07±0.10a,X	1.07±0.10a,X
4	W	0.005±0.000a,X	0.004±0.001a,X	0.98±0.06a,X	1.08±0.05a,X
	D	0.001±0.000a,Y	0.001±0.001a,Y	1.01±0.01b,X	1.21±0.10a,X
8	W	0.010±0.003a,X	0.005±0.005a,X	0.91±0.02a,X	0.92±0.06a,X
	D	0.000±0.000a,Y	0.001±0.000a,Y	0.90±0.11a,X	0.88±0.04a,X
12	W	0.001±0.001a,X	0.004±0.001b,X	0.80±0.01a,X	0.82±0.04a,X
	D	0.001±0.000a,X	0.001±0.001a,Y	0.73±0.06a,X	0.74±0.06a,X
15	W	0.002±0.001a,X	0.003±0.002a,X	0.75±0.13b,X	0.85±0.06a,X
	D	0.000±0.000a,Y	0.001±0.001a,X	0.64±0.02a, X	0.72±0.04a,Y
18	W	0.003±0.002a,X	0.002±0.001a,X	0.62±0.03b, X	0.69±0.04a,X
	D	0.000±0.000 a,Y	0.000±0.000 a,Y	0.56±0.04a, X	0.62±0.04a,X

a,b=Different lowercase letters in the same row indicate significant differences (p<0.05) between samples in the same muscle. X,Y=Different capital letters in the same column indicate the significant difference (p<0.05) between two muscle in the same sample type. TPA=Texture profile analysis

Hardness is the force needed to create a given deformation in muscle and greatly depends on the structure of the connective tissue [37]. It was significantly reduced during storage time indicating that VP alone cannot maintain the firmness of fillet on a stable level. This fact might be due to the low pH causing muscle protein denaturation [38]. Hardness values of white muscles were significantly (p<0.05) higher than those of dark muscles. These differences between the hardness of the two muscles could be due to the differences in proximate composition and enzymatic activity. In addition, dark muscle had a higher proteolytic activity than white muscle [39]. Both white and dark muscles from control were significantly (p<0.05) softer than treated samples throughout the storage time, indicating that the use of oregano extract in combination with VP can avoid softening and improve the protein denaturation of fish muscles during storage.

Springiness is the height recovering property of the fish muscle between the end of the first and the beginning of the second compressions [40]. Springiness decreased in all samples over time and might be caused by cathepsins [41]. At day 0, the springiness value of white muscle was significantly (p<0.05) lesser than dark muscle. However, no significant differences (p>0.05) on the springiness of white and dark muscle were observed in the rest of the storage period. No significant differences in springiness values of both treatments were observed either. These results indicated that oregano extract had a major effect on reducing the springiness of only dark muscle.

Adhesiveness is the work needed to pull the compressing plunger from sample [42]. A significant decrease in the adhesiveness of white muscle from treated and control was observed until 4 and 8 days of storage, respectively, followed by a marked increase in these values on the next testing day. The same tendency was observed for dark muscle, where adhesiveness increased markedly on days 4 and 8 in treated and control, respectively. Significant (p<0.05) differences in adhesiveness were observed between the dark and white muscles of the treated samples only at 4 and 8 days of the storage, respectively, where each muscle of the treated groups was significantly (p<0.05) higher than control. However, adhesiveness values of dark muscle were significantly higher than those of white muscle from each treated and control groups at 4 and 8 days, respectively. The increase in adhesiveness might be due to the microbial growth resulting in proteins degradation [43]. For the rest of the storage period, no significant (p>0.05) differences on adhesiveness values were observed between treatments as well as both muscles type. Thus, the combination of the use of oregano extract with VP seemed to improve the adhesiveness of frigate tuna muscles on the earlier stage of storage. In addition, oregano extract seemed to have a better effect on dark muscle compared to white muscle.

The elasticity of the fish muscles, given in resilience, was significantly (p<0.05) higher in the white muscle than in the dark muscle throughout the experiment. Regardless of muscle forms, changes in resilience with time were not significant (p>0.05) between both treatments. Therefore, the combined application of oregano and VP did not affect the elasticity of the white and dark muscles.

Chewiness is the work required to masticate a solid food to a steady state of swallowing [37]. Chewiness was affected in all samples and decreased with storage time. No significant (p>0.05) differences in chewiness of white and dark muscle were observed throughout the storage. Statistical data showed that treatment did not affect the chewiness of both muscles, indicating that the application of oregano extract in combination with VP cannot enhance the chewiness of both white and dark muscles.

Consequently, VP alone cannot enhance texture parameters of frigate tuna muscles. The combined use of VP and oregano extract can alter the texture parameters and had the major effect on only hardness, adhesiveness, and springiness of both white and dark muscles of frigate tuna fillets.

## Conclusion

Based on the sensory assessment, the shelf life of both frigate tuna muscles was 12 days for the control and 18 days for the treated groups. The microbiological results showed that the microbial load was not affected by the type of muscle during the refrigerated (3±1°C) storage since skin and white muscle provide together a protective environment for dark muscle as in the intact fillet or even in the whole fish. It can be concluded that the test portion from the white/dark muscle was sufficient to estimate the microbiological quality of the analyzed product.

The effect of the combination of oregano extract with VP on white and dark muscles of frigate tuna fillet stored as intact fillets without skin removal revealed lower in the chemical and microbiological indices due to the antioxidant and antimicrobial properties of the extract that enhanced texture parameters. Such combination can further delay the deterioration of fish freshness as they have a promising cumulative effect that can be used by the seafood processing industry to extend the shelf life of seafood products.

## Authors’ Contributions

TL, TMH, and FO: Conceived and designed study. TL, YU, and ARK: Collected samples and performed the experiments. TL: Performed the statistical analysis. TL, TMH, and FO: Drafting and revising the manuscript critically for important intellectual content. All authors read and approved the final manuscript.
